# Factors associated with mental health and quality of life during the COVID-19 pandemic in Brazil

**DOI:** 10.1192/bjo.2021.62

**Published:** 2021-05-14

**Authors:** Luciano Magalhães Vitorino, Gerson Hiroshi Yoshinari Júnior, Gabriela Gonzaga, Isabela Faria Dias, João Pedro Lambert Pereira, Isabella Marum Góes Ribeiro, Alex Bacadini França, Faten Al-Zaben, Harold G. Koenig, Clarissa Trzesniak

**Affiliations:** Faculty of Medicine of Itajubá, Afya Group, Brazil; Faculty of Medicine of Itajubá, Afya Group, Brazil; Faculty of Medicine of Itajubá, Afya Group, Brazil; Faculty of Medicine of Itajubá, Afya Group, Brazil; Faculty of Medicine of Itajubá, Afya Group, Brazil; Faculty of Medicine of Itajubá, Afya Group, Brazil; Federal University of São Carlos – UFSCar, Laboratory of Human Development and Cognition – LADHECO, Brazil; Division of Psychiatry, Department of Medicine, King Abdulaziz University, Saudi Arabia; Psychiatry & Behavioral Sciences, Duke University Medical Center, USA; and Department of Medicine, King Abdulaziz University, Saudi Arabia; Faculty of Medicine of Itajubá, Afya Group, Brazil

**Keywords:** COVID-19, quarantine, depression, mental health, quality of life

## Abstract

**Background:**

Although mental distress and quality of life (QoL) impairments because of the pandemic have increased worldwide, the way that each community has been affected has varied.

**Aims:**

This study evaluated the impact of social distancing imposed by coronavirus disease-2019 (COVID-19) on Brazilians’ mental health and QoL.

**Method:**

In this cross-sectional community-based online survey, data from 1156 community-dwelling adults were gathered between 11 May and 3 June 2020. We examined independent correlates of depression, anxiety and QoL, including sociodemographic and clinical characteristics, optimism/pessimism and spiritual/religious coping. Dependent variables were assessed using the 9-item Patient Health Questionnaire for depressive symptoms, the 7-item Generalized Anxiety Disorder Scale for anxiety symptoms, and the World Health Organization Quality of Life-BREF for QoL. Correlates of depressive and anxiety disorder were estimated using logistic regression.

**Results:**

There were high levels of depressive symptoms (41.9%) and anxiety symptoms (29.0%) in participants. Negative spiritual/religious coping was positively correlated with depressive disorder (odds ratio (OR) = 2.14 95% CI 1.63–2.80; *P* < 0.001) and with anxiety disorder (OR = 2.46 95% CI 1.90–3.18; *P* < 0.001), and associated with worse social and environmental QoL (*P* < 0.001). Healthcare professionals were less likely to have depressive symptoms (OR = 0.71, 95% CI 0.55–0.93; *P* < 0.001). Participants with friend/family with COVID-19 scored lower on psychological and environmental QoL (*P* < 0.05). Participants with a longer duration of social isolation were less likely to experience anxiety disorder (OR = 0.99, 95% CI 0.98–0.99; *P* = 0.004).

**Conclusions:**

We found high levels of depressive and anxiety symptoms and low levels of QoL in Brazil, which has become a pandemic epicentre. Several characteristics were associated with negative mental health symptoms in this study. This information may contribute to local health policies in dealing with the mental health consequences of COVID-19.

## Background

Coronavirus disease-2019 (COVID-19) is a disease caused by infection with the SARS-COV-2 virus, a member of the coronavirus family.^1^ COVID-19 can manifest clinically from being asymptomatic to developing acute respiratory distress syndrome, a potentially fatal condition.^1,2^ Given its highly contagious nature, the World Health Organization (WHO) declared COVID-19 a pandemic in January 2020.^3^ As there is no specific treatment for COVID-19, in an attempt to slow down the spread of the disease, many public health authorities have recommended social distancing and even quarantine in some situations.^4^ The impact of this strategy of social isolation on mental health and quality of life (QoL) is unknown since the world has not experienced a pandemic of this extent in the last century.

The influence of catastrophic natural events on public mental health has been previously described.^5^ Hurricane Ike led to major depressive disorder in 5% of the affected population assessed 1 month afterwards.^6^ The SARS epidemic in 2003, caused by SARS-COV-1, also resulted in high psychological distress among survivors.^7^ Self-reported psychological distress and loneliness has grown during the current COVID-19 pandemic in the USA, when 2018 and 2020 were compared.^8^ The same effects have been observed in China, where a quarter of all mental health patients in institutions reported issues related to COVID-19.^9,10^ A high percentage of healthcare professional also report mental health problems.^11^ Unfortunately, mental health support to the general population has often been lacking.^9,12^

## Situation in Brazil

Although the rise in mental distress and QoL impairments because of the pandemic seem to be the rule, the way that each community has been affected has varied.^12^ Regional differences should be taken into consideration since local aspects of culture (such as religion, economic situation, unemployment) influence mental health.^4,9,10^ In Brazil, the first case of COVID-19 was officially detected on 25 February 2020. Although the country had reached its highest death rates during the first wave of the pandemic, the federal government did not develop a national plan to combat the pandemic and did not impose a lockdown until later.^13^ Despite this, many state governors and mayors decreed that people should stay at home, and social clubs, schools and universities were closed. Brazilians were instructed to adopt protective measures, such as social distancing, hand washing and wearing masks.

Four months after the beginning of the COVID-19 pandemic, Brazil became an epicentre of this disease with one of the highest case and death rates. Although some states and large cities have achieved a plateau in terms of new cases, the virus is now beginning to reach small and medium-sized cities where the healthcare system is even more fragile.^14^ Even with highly concerning daily statistics and considerable underreporting of cases, the federal government's response has been less than optimum in facing this health emergency.^15^ Feelings of fear, insecurity, loss, and inefficiency in combating this pandemic has adversely affected the mental health and QoL of many Brazilians.

The present study seeks to examine the association between social distancing and other demographic and clinical characteristics and Brazilians’ self-reported mental health and QoL.

## Method

A cross-sectional community-based online survey was conducted during the heart of the COVID-19 pandemic in Brazil between 11 May and 3 June 2020. The Research Ethics Committee of the Faculty of Medicine of Itajubá, Brazil, approved this study (#4,010,466). All participants gave informed consent online. All procedures were carried out under Brazil's ethics regulations and the 1964 Helsinki Declaration.

### Data collection, location and participants

Data collection was carried out using an online electronic form, which was prepared by using the Google Forms application. Data collection started 3 months after Brazilian law was instituted to ensure quarantining and social distancing as a response to the new coronavirus. A link (https://forms.gle/L669qRyRDM4w2wdk6) to the questionnaire was sent through social networks (Facebook, Instagram and WhatsApp). A total of 1156 volunteers from 22 out of the 27 Brazilian states and 196 cities completed the questionnaire. Participants were required to be 18 years and over, in quarantine for at least 15 days (except health professionals), Brazilian or naturalised citizens, and reside in Brazilian territory. Participants with more than 20% of missing data were excluded.

### Dependent variables

The Patient Health Questionnaire (PHQ-9)^16^ was used to identify depressive symptoms in the past 2 weeks. The PHQ-9 asks about the nine symptoms of major depression disorder as required by the DSM-5. Responses to each item are rated on a 4-point Likert scale, ranging from 0 (‘not at all’) to 3 (‘almost every day’). Higher overall scores indicate increased levels of depressive symptoms, which can vary from 0 to 27.^16^ A cut-off score of ≥ 10 points indicates the presence of significant depressive symptoms (‘moderate’, ‘moderately severe’ or ‘severe’).^16^ In the present study, the instrument showed excellent internal reliability (α = 0.91).

The 7-item Generalized Anxiety Disorder Scale (GAD-7) was used to assess symptoms experienced over the past 2 weeks related to generalized anxiety disorder.^17^ Response options for each of the seven items are rated on a 4-point Likert scale, ranging from 0 (‘not at all’) to 3 (‘almost every day’). Higher overall scores indicate more frequent and severe anxiety symptoms, with total scores ranging from 0 to 21. We adopted a cut-off score of ≥10 to identify anxiety disorder (‘moderate’ or ‘severe’).^17^ The GAD-7 demonstrated solid internal consistency with a Cronbach's alpha = 0.94.

The World Health Organization Quality of Life-BREF (WHOQOL-BREF) is a QoL scale that contains 26 items responded to on a 5-point Likert scale from 1 to 5.^18^ The following four domains are assessed by this measure: physical, psychological, social relationships and environmental. Higher scores indicate better perceptions of QoL. There is no cut-off score for ‘case’ identification.^18^ The internal consistency of each of the four domains were: physical health (α = 0.80), psychological (α = 0.81), social relationships (α = 0.77) and environmental (α = 0.79).

### Independent variables

The variables below were selected as they may affect the outcomes of mental health and QoL domains during the COVID-19 pandemic.^19,20^

Sociodemographic information collected were age, gender (male or female) and marital status (single, married or divorced). Also determined were whether any family member or friend was diagnosed with COVID-19 (yes or no), and whether the person was a healthcare professional (yes or no). Physical health problems were also enquired about, including having chronic disease (yes or no), use of medication daily (yes or no), use of controlled medications (anxiolytics/antidepressants; yes or no), having recently been seen in a primary healthcare unit (yes or no), and having consulted a psychologist (yes or no). Participation in regular physical activity (at least three times a week; yes or no) was also assessed. Finally, participants were asked to categorise themselves into whether they were religious and/or spiritual by responding to the question: ‘How religious and/or spiritual are you.’ Possible answers were high religiosity and high spirituality, high religiosity and low spirituality, low religiosity and high spirituality, and low religiosity and low spirituality. This question is commonly used to determine self-identification as religious or spiritual, and has been used in previous studies in Brazil.^21^

The period of social isolation was assessed with a single question: ‘How long have you been in social isolation (in days)?’

Optimism and pessimism were assessed by the Revised Life Orientation Test (LOT-R), validated by Bandeira et al in a Brazilian population.^22^ The LOT-R has ten items: three assessing optimism, three examining pessimism and four filler items that are not included in the analysis. Each item is classified on a 5-point Likert scale ranging from 0 (‘strongly disagree’) to 4 (‘strongly agree’). The total scale score is calculated by summing the optimism score subscale and the reverse-scored pessimism score subscale.^22^ The LOT-R had acceptable internal reliability in the present sample (optimism α = 0.78 and pessimism α = 0.83).

Spiritual/religious coping (SRC) was assessed by the Brief Scale for Spiritual/Religious Coping (SRCOPE-14) for assessing SRC domains.^23^ This scale was originally developed by Pargament et al,^23^ and has been validated in a Brazilian population.^24^ The SRCOPE-14 assesses two dimensions of religious coping: positive SRC (PSRC; items 1–7) and negative SRC (NSRC; items 8–14). PSRC has items related to spirituality/religiosity as a source of love, care, strength, help, purification and positive reframing of the stressor. The NSRC contains items that assess spiritual or religious conflict (i.e. feelings of being punished by God, deserted by one's faith community, feeling that God cannot help, and so forth). For each item, responses are rated on a 5-point Likert scale, ranging from 1 (‘not even a little/not applicable’) to 5 (‘a lot/very applicable’). Averages for each item on the subscale were calculated and then those averaged, with total scores varying from 1 to 5. Higher scores represent greater SRC (positive or negative).^24^ The two dimensions demonstrated acceptable internal consistency (PSRC α = 0.94 and NSRC α = 0.88).

### Data analyses

The data were analysed using the software Statistical Package for Social Sciences - SPSS 26 (SPSS Inc.). Descriptive statistics were performed with frequencies for sociodemographic and health characteristics, including the prevalence of significant anxiety and depressive symptoms. The optimism and pessimism scores, SRC scores and dependent variables (QoL, depressive symptoms and anxiety disorder) were presented with means, standard deviations (s.d.) and 95% confidence intervals (95% CI). Student's *t*-test and one-way ANOVA were used to compare the dependent variable means, grouping by the independent variables (for example gender marital status, education). Pearson's correlation was performed between continuous independent variables (for example age, quarantine duration in days, optimism, pessimism and SRC-14) and dependent variables.

Logistic regression models were used to explore the associations between independent variables and significant depressive symptoms and significant anxiety symptoms. Multivariate general linear models (GLM) were used to assess the effects of the independent variables (for example gender, being a healthcare professional) on the four domains of the WHOQOL-BREF. The multivariate GLM were used to controlled for covariates when examining continuous outcomes. Multivariate logistic regression models and GLM included only those independent variables that reached a *P* < 0.10 in bivariate analyses. For all analyses, alpha level for statistical significance was set at *P* < 0.05 (two-tailed).

## Results

From the 1167 participants approached for the study, 1156 (99%) completed all questionnaire items. Table 1 presents the sociodemographic characteristics of the participants. The mean age of participants was 37.6 years (s.d. = 14.0), 27.7% had a friend or family member with COVID-19, and 34.3% were healthcare professionals. The average time spent in social isolation at the time of completing the survey was 46.5 days (95% CI 45.3–47.7).

**Table 1:** Sociodemographic and health characteristics of sample (*n* = 1156)

Variables	Value
Age, mean (s.d.)	37.6 (14.0)
Gender, *n* (%)	
Male	351 (30.4)
Female	804 (69.6)
Marital status, *n* (%)	
Single	483 (41.7)
Married/living together	566 (49.0)
Divorced	107 (9.3)
Friend/Family with COVID-19, *n* (%)	
Yes	323 (27.9)
No	833 (72.1)
Healthcare professional status, *n* (%)	
Yes	396 (34.3)
No	760 (65.7)
Chronic disease, present, *n* (%)	
Yes	274 (23.7)
No	882 (76.3)
Daily medication, *n* (%)	
Yes	537 (46.5)
No	619 (53.5)
Controlled medication, *n* (%)	
Yes	264 (22.8)
No	892 (77.2)
Physical activity^^a^,^b^^, *n* (%))	
Yes	388 (33.6)
No	768 (66.4)
Primary Care unit use during the pandemic, *n* (%)	
Yes	138 (11.9)
No	1018 (88.1)
Consultation with a doctor or psychologist during the pandemic, *n* (%)	
Yes	288 (24.9)
No	868 (75.1)
Spirituality/religiosity, *n* (%)	
High religiosity and high spirituality	323 (27.9)
High religiosity and low spirituality	98 (8.5)
Low religiosity and high spirituality	451 (39.0)
Low religiosity and low spirituality	284 (24.6)
Anxiety symptoms (GAD-7 ≥ 10), *n* (%)	
Yes	335 (29.0)
No	821 (71.0)
Depressive symptoms (PHQ-9 ≥ 10), *n* (%)	
Yes	484 (41.9)
No	672 (58.1)

GAD-7: General Anxiety Disorder-7; PHQ-9: Patient Health Questionnaire-9.

a. During social isolation.

b. Regular (at least three times a week).

Regarding categories of spirituality and religiousness, 27.9% indicated they were both high religiosity and high spirituality, 8.5% said they were high religiosity and low spirituality, 39.0% indicated they were low religiosity and high spirituality; and 24.6% reported they were low religiosity and low spirituality. Although religious affiliation was not assessed, the 2010 Brazilian census found that religious affiliation at the country level was 64% Catholic, 22% Protestant, 2% Spiritism, 3% other, 8% none.

The frequency of anxiety disorder (GAD-7 ≥ 10 points) and depressive disorder (PHQ-9 ≥ 10 points) were 29.0% and 41.9%, respectively. Table 2 presents the average scores for all mental health outcomes.

**Table 2:** Mean scores^a^ for quality of life, depressive symptoms, anxiety, optimism, pessimism and spiritual/religious coping (*n* = 1156)

Variables	Mean (s.d.), 95% CI
World Health Organization Quality of Life-BREF	
Physical health	14.7 (2.7), 14.6–14.9
Psychological	14.1 (2.8), 13.9–14.3
Social relationships	13.9 (3.5), 13.6–14.1
Environment	14.6 (2.45), 14.5–14.8
Depression (Patient Health Questionnaire-9)	
Total score	9.2 (6.6), 8.8–9.6
Anxiety (General Anxiety Disorder-7)	
Total score	7.0 (6.1), 6.7–7.4
Optimism	
Total score	8.6 (2.6), 8.4–8.7
Pessimism	
Total score	7.5 (2.6), 7.4–7.6
Positive spiritual/religious coping	
Total score	2.7 (1.2), 2.7–2.8
Negative spiritual/religious coping	
Total score	1.2 (0.5), 1.2–1.3

a. Mean score was adjusted for age, healthcare professional, days of social isolation, optimism, pessimism, positive and negative spiritual religious coping.

### Bivariate analyses

Table 3 presents the bivariate associations between categorical independent variables and continuous scores on depressive symptoms, anxiety symptoms and QoL domains. On depressive symptoms, healthcare professionals had lower scores than non-healthcare professionals (*P* = 0.002); patients with chronic diseases had higher scores than those without (*P* = 0.027), as did participants who took daily medication (*P* = 0.017), those who took controlled medications (*P* = 0.005), and those seen at healthcare units (*P* = 0.03). In contrast, those who are engaged in regular physical activities during social isolation experienced lower depressive symptoms (*P* = 0.038). With regards to anxiety scores on the GAD-7, participants who took controlled medication had higher scores than those who did not (*P* = 0.023), as did individuals who consulted with a psychiatrist or psychologist (*P* = 0.046).

**Table 3:** Bivariate associations between categorical independent variables and depressive symptoms, anxiety and quality of life domains (on the World Health Organization Quality of Life-BREF) (*n* = 1156)

Variables	PHQ-9, mean (s.d.)	GAD-7, mean (s.d.)	Physical, mean (s.d.)	Psychological, mean (s.d.)	Social relationships, mean (s.d.)	Environment, mean (s.d.)
Gender						
Male	9.2 (6.6)	7.5 (6.5)	14.8 (2.6)	14.4 (2.7)	14.1 (3.6)	14.8 (2.3)
Female	9.1 (6.5)	6.8 (5.9)	14.7 (2.6)	14.0 (2.8)	13.7 (3.4)	14.6 (2.5)
*P*	0.785	0.060	0.557	0.057	0.165	0.115
Marital status						
Single	9.6 (6.4)	7.2 (5.9)	14.6 (2.6)	13.9 (2.8)	13.6 (3.5)	14.5 (2.4)
Married/living together	8.9 (6.7)	6.9 (6.1)	14.8 (2.7)	14.2 (2.8)	13.9 (3.5)	14.6 (2.4)
Divorced	8.3 (6.2)	7.0 (7.0)	14.7 (2.6)	14.7 (2.9)	14.5 (3.4)	14.9 (2.6)
*P*	0.100	0.760	0.478	0.019	0.071	0.396
Friend/family with COVID-19						
Yes	9.1 (6.3)	6.7 (5.8)	14.7 (2.5)	14.2 (2.8)	13.6 (3.5)	14.4 (2.4)
No	9.2 (6.6)	7.1 (6.2)	14.7 (2.7)	14.1 (2.8)	13.9 (3.5)	14.7 (2.5)
*P*	0.743	0.273	0.676	0.534	0.169	0.022
Healthcare professionals						
Yes	8.3 (6.2)	6.6 (6.3)	15.0 (2.4)	14.6 (2.8)	14.1 (3.5)	15.0 (2.3)
No	9.6 (6.7)	7.2 (6.0)	14.6 (2.5)	13.9 (2.8)	13.7 (3.5)	14.5 (2.5)
*P*	0.002	0.147	0.002	<0.001	0.108	0.001
Chronic disease						
Yes	10.0 (7.0)	7.5 (6.5)	14.7 (2.5)	13.9 (2.8)	13.5 (3.4)	14.5 (2.3)
No	8.9 (6.4)	6.9 (6.0)	14.7 (2.7)	14.2 (2.8)	13.9 (3.5)	14.7 (2.5)
*P*	0.027	0.190	0.639	0.179	0.069	0.441
Daily medication						
Yes	9.6 (6.7)	7.4 (6.2)	14.5 (2.6)	13.9 (2.9)	13.6 (3.3)	14.6 (2.4)
No	8.7 (6.4)	6.7 (6.0)	14.8 (2.7)	14.3 (2.7)	14.0 (3.6)	14.7 (2.4)
*P*	0.017	0.059	0.045	0.013	0.047	0.355
Controlled medication						
Yes	10.2 (6.9)	7.8 (6.3)	14.6 (2.7)	13.7 (2.9)	13.5 (3.4)	14.6 (2.6)
No	8.8 (6.4)	6.8 (6.0)	14.7 (2.6)	14.2 (2.8)	13.9 (3.5)	14.6 (2.4)
*P*	0.005	0.023	0.433	0.012	0.072	0.688
Physical activity^^a^,^b^^						
Yes	8.6 (6.4)	6.6 (5.8)	14.9 (2.6)	14.3 (2.7)	13.9 (3.4)	14.7 (2.3)
No	9.4 (6.6)	7.2 (6.3)	14.6 (2.6)	14.0 (2.8)	13.8 (3.5)	14.6 (2.5)
*P*	0.037	0.100	0.151	0.143	0.618	0.256
Primary Care unit use during the pandemic^a^						
Yes	10.4 (6.9)	7.8 (6.3)	14.5 (2.9)	13.8 (2.9)	13.3 (3.8)	14.4 (2.8)
No	9.0 (6.5)	6.9 (6.1)	14.7 (2.6)	14.2 (2.8)	13.9 (3.4)	14.7 (2.4)
*P*	0.030	0.116	0.443	0.187	0.066	0.210
Consultation with a doctor or psychologist during the pandemic^a^						
Yes	9.7 (6.9)	7.7 (6.7)	14.8 (2.5)	14.3 (2.9)	13.9 (3.6)	14.7 (2.4)
No	8.9 (6.4)	6.8 (5.9)	14.7 (2.7)	14.1 (2.8)	13.8 (3.4)	14.6 (2.4)
*P*	0.092	0.046	0.466	0.196	0.616	0.346
Spirituality/religiosity^a^						
High religiosity and high spirituality	9.2 (6.5)	7.2 (6.1)	14.6 (2.6)	14.2 (2.8)	14.1 (3.3)	14.6 (2.5)
High religiosity and low spirituality	8.0 (6.2)	5.8 (5.2)	15.4 (2.4)	14.6 (2.7)	14.3 (3.3)	14.9 (2.4)
Low religiosity and high spirituality	9.2 (6.5)	7.1 (6.3)	14.8 (2.6)	14.3 (2.8)	14.0 (3.6)	14.8 (2.4)
Low religiosity and low spirituality	9.5 (6.8)	7.0 (5.9)	14.6 (2.8)	13.7 (2.8)	13.1 (3.6)	14.4 (2.5)
*P*	0.269	0.251	0.039	0.035	0.002	0.146

PHQ-9, Patient Health Questionnaire-9; GAD-7, General Anxiety Disorder-7.

a. During quarantine.

b. Regular (at least three times a week).

Healthcare professionals reported significantly higher QoL physical (*P* = 0.002), psychological (*P* < 0.001) and environment (*P* = 0.001) scores. In contrast, participants taking daily medication scored lower on QoL physical (*P* = 0.045)), psychological (*P* = 0.013) and social relationships (*P* = 0.047) scores. Moreover, participants who took controlled medication and had friend/family relatives with COVID-19 had lower psychological (*P* = 0.012) and environment (*P* = 0.022) scores.

There were significant differences based on spiritual/religious categories on the physical (*F* = 2.800; *P* = 0.039), psychological (*F* = 2874; *P* = 0.035), and social relationships (*F* = 5.119; *P* = 0.002) QoL domain scores.

After Bonferroni correction, those who were religious but not spiritual scored significantly higher on the physical health QoL domain compared with those who were both spiritual and religious (*P* = 0.048) and those who were neither religious nor spiritual (*P* = 0.043). Those who were neither religious nor spiritual also scored lower on the QoL social relationships domain than those who were both religious and spiritual (*P* = 0.008), those who were religious but not spiritual (*P* = 0.035) and those who were spiritual but not religious (*P* = 0.005).

Bivariate correlations between dependent variables and age, days of social isolation, optimism, pessimism and SRC are presented in Table 4. With regard to SRC, NSRC scores were positively related to depressive symptoms and anxiety symptoms, and with worse physical WHOQOL scores, ranging from *r* = 0.246 to *r* = 0.260, all *P* < 0.001. Likewise, NSRC was negatively correlated with psychological (*r* = −0.283, *P* < 0.001) and environment (*r* = −0.293, *P* < 0.001) WHOQOL domain scores.

**Table 4:** Correlations between dependent variables and age, days of social isolation, optimism, pessimism, and spiritual/religious coping (*n* = 1156)

Variables	Age	Period of social isolation in days	Optimism	Pessimism	Positive spiritual/ religious coping	Negative spiritual/ religious coping
PHQ-9	−0.055	−0.037	−0.092**	−0.025	0.027	0.260**
95% CI	−0.113 to 0.002	−0.091 to 0.018	−0.151 to −0.034	−0.085 to 0.033	−0.031 to 0.084	0.190 to 0.317
GAD-7	−0.045	−0.105**	−0.045	−0.044	0.092**	0.246**
95% CI	−0.100 to 0.010	−0.126 to −0.042	−0.107 to 0.016	−0.101 to 0.011	0.032 to 0.151	0.185 to 0.298
*WHOQOL-BREF*						
Physical	0.016	0.016	0.054	0.026	0.003	−0.255**
95% CI	−0.043 to 0.073	−0.041 to 0.071	−0.004 to 0.113	−0.031 to 0.086	−0.055 to 0.061	−0.312 to −0.193
Psychological	0.042	0.009	0.110**	0.051	0.101**	−0.283**
95% CI	−0.016 to 0.100	−0.051 to 0.070	0.051 to 0.169	−0.004 to 0.112	0.041 to 0.155	−0.342 to −0.221
Social relationships	0.027	−0.016	0.089**	0.013	0.065*	−0.168**
95% CI	−0.031 to 0.082	−0.079 to 0.043	0.026 to 0.148	−0.040 to 0.074	0.004 to 0.122	−0.235 to −0.107
Environment	0.026	0.008	0.079**	0.047	−0.070*	−0.293**
95% CI	−0.032 to 0.078	−0.050 to 0.065	0.016 to 0.138	−0.012 to 0.105	−0.130 to −0.010	−0.352 to −0.236

PHQ-9, Patient Health Questionnaire-9; GAD-7, General Anxiety Disorder-7; WHOQOL, World Health Organization Quality of Life.

*P < 0.05; **P < 0.01.

## Multivariate analyses

### Depression and anxiety

Multivariate analyses demonstrated that NSRC scores were positively correlated with a significant level of depressive symptoms (OR = 2.14, 95% CI 1.63–2.80; *P* < 0.001) (Supplementary Table 1 available at https://doi.org/10.1192/bjo.2021.62). In contrast, being a healthcare professional (OR = 0.71, 95% CI 0.55–0.93; *P* < 0.001) and being more optimistic (OR = 0.94, 95% CI 0.90–0.99; *P* = 0.018) were characteristics that meant participants were less likely to report depressive symptoms.

Regarding anxiety disorder symptoms, multivariate analyses indicated that those with NSRC scores were also more likely to have significant anxiety symptoms (OR = 2.46, 95% CI 1.90–3.18; *P* < 0.001). Participants with longer social isolation duration were surprisingly less likely to report high anxiety symptoms (OR = 0.99, 95% CI 0.98–0.99; *P* = 0.004).

### QoL domains

The multivariate analyses examining predictors of QoL domains are provided in Supplementary Table 2. MANOVA indicated that healthcare professionals had higher psychological (*F* = 6.993; *P* = 0.008) and environmental (*F* = 5.458; *P* = 0.020) QoL scores. Participants who had friend/family with COVID-19 scored lower on psychological (*F* = 3.796; *P* = 0.010), social relationships (*F* = 2.823; *P* = 0.038) and environmental (*F* = 8.95; *P* = 0.004) QoL domains. Not surprisingly, depressive symptoms were associated with lower QoL scores on all domains. With regard to SRC, PSRC was associated with higher scores on psychological (*F* = 49.351; *P* < 0.001) and social relationships (*F* = 10.585; *P* = 0.001) QoL domains. NSRC, in contrast, was associated with significantly lower scores on social relationships (*F* = 10.596; *P* < 0.001) and environmental (*F* = 51.245; *P* < 0.001) QoL domains.

## Discussion

### Main findings

The present study examined the relationship between mental health and QoL domains among Brazilians during the heart of the COVID-19 pandemic. Lack of cooperation between government actions and health professionals and scientists contributed to the pandemic's worsening, which has had seriously affected the Brazilian population.^14,15^ Our results here revealed a high frequency of depressive symptoms, anxiety symptoms and impaired QoL among Brazilians at this crucial time during the COVID-19 pandemic. Participants with higher NSRC (such as a feeling of abandonment by God, Divine punishment, doubts about God's love) were more likely to report high levels of depressive and anxiety symptoms. Healthcare professionals and more optimistic participants, in turn, were less likely to report significant levels of depressive and anxiety symptoms. Surprisingly, longer time spent in quarantine (social isolation) was associated with a slightly lower likelihood of reporting significant anxiety symptoms.

Regarding QoL, healthcare professionals and participants with higher levels of PSRC (for example greater connection with God, love of God, putting plans into action with God's help) experienced better QoL. With regards to the higher QoL in health professionals, it could be that physicians have a higher income and more control of their schedule compared with others, thus increasing their QoL. In contrast, those who had a friend or family member with COVID-19, depressive symptoms, anxiety symptoms or engaged in NSRC scored lower on QoL domains.

To our knowledge, this is the first study to use psychometrically valid measures to assess mental health, SRC and QoL during the COVID-19 pandemic quarantine period in Brazil. This allows for a comparison with similar situations in other countries, in addition to advancing our understanding of the quarantine's impact on the mental health and QoL of Brazilians.

### Comparison with findings from other studies and other parts of the world

A report by the WHO published in 2015 found that the global prevalence of significant depressive and anxiety symptoms was 4.4% and 3.6%, respectively.^25^ In 2017, the Brazilian population estimate was 5.8% for significant depressive symptoms and 9.3% for significant anxiety symptoms.^25^ In comparison with the present sample during the heart of the COVID-19 pandemic, this represents an increase of 722% (from 5.8% to 41.9%) in significant depressive symptoms and a 312% increase (from 9.3% to 29%) in significant anxiety symptoms. Recent Brazilian online surveys reported a similar increase in significant anxiety symptoms (from 39.7% to 81.9%) and depressive symptoms (from 40.4% to 68.0%) during the COVID-19 pandemic.^26–28^ About 19.4% to 21.5% of Brazilians reported severe/extreme symptoms of anxiety and depression, respectively, during the COVID-19 pandemic.^29^

The COVID-19 pandemic has been reported to affect the mental health of the population in several countries. For example, a global review with 66 studies (*n* = 221 970) identified a prevalence of 31.4% for depression and 31.9% for anxiety during the COVID-19 pandemic.^30^ In China, significant depressive and anxiety symptoms among the general public were reported to be 26% and 22%, respectively, and among healthcare professionals were 31% and 40%, respectively.^31^ Likewise, in the USA, significant depressive and anxiety symptoms were reported to be present in 43.3% and 45.4%, respectively.^32^ A recent meta-analysis identified a prevalence of 33.7% for significant depressive symptoms during the pandemic (95% CI 27.5–40.6; 14 studies, *n* = 44 531), and of 31.9% for significant anxiety symptoms (95% CI 27.5–36.7; 17 studies, *n* = 63 439).^33^ The threat of COVID-19 infection and the increased mortality associated with it, as well as the forced social isolation, have generated much stress in populations worldwide.^34,35^ This distress may be enhanced by the death of loved ones, loss of jobs and income, negative attention presented by mass media, restrictions on mobility and insufficient efforts by governments to combat the pandemic.^15,36^

### Impact of religious beliefs

Brazilians are a very religious people compared with populations in many other countries.^37^ There is evidence that religiosity/spirituality can have a positive impact on mental health and QoL outcomes.^21,38,39^ This, however, is not always the case. Religious or spiritual struggles, such as feeling punished or abandoned by God or their faith community, can trigger mental health problems.^40^ SRC is frequently used in stressful situations, such as during the COVID-19 pandemic. Positive forms of SRC are more prevalent and associated with better mental health outcomes.^23^ Negative forms of SRC are a warning sign, as they are often associated with worse mental health.^23^ Our findings reinforce these findings. During the pandemic, Brazilians with NRSC were more likely to have significant levels of depressive and anxiety symptoms. Findings from the USA and Turkey also indicate that negative forms of SRC are associated with worse mental health during the COVID-19 pandemic.^41,42^

### Healthcare professionals

Contrary to expectations, healthcare professionals, who represented more than one-third of our study participants, experienced a lower likelihood of having significant depressive symptoms compared with other non-healthcare professionals. Other research has suggested significantly increased mental health problems among healthcare professionals during the COVID-19 pandemic.^11,35^ A possible explanation for the results reported in the present study is that not all healthcare professionals were working on the COVID-19 frontline. Furthermore, healthcare professionals’ knowledge may give them a sense of control that has helped to alleviate depressive symptoms during the pandemic.^43^ A lower level of depressive symptoms among healthcare professionals could also be explained by the fact that they see their work as serving a higher purpose in helping to save lives, which may also be reinforced by their religious belief system.

### Optimism

We also found that participants who were more optimistic about the future were less likely to have significant levels of depressive symptoms. An online survey during the COVID-19 pandemic with Dutch and Belgian participants had similar findings. Positive personality traits such as optimism may protect against negative mental health consequences (i.e. fear of the coronavirus, depression and anxiety).^44^ An optimistic view of life has been found in many different cultures to be associated with a lower risk of depression.^45^

### Quarantine

Surprisingly, however, a longer quarantine duration was associated with a lower likelihood of high anxiety symptoms in the present study. This result contrasts with findings from other studies reporting that the longer a quarantine lasts the higher the prevalence of psychological disorders. Each individual manages stress, feelings, and fears in a unique way, and the ultimate outcome will depend on coping mechanisms and underlying resilience. Factors such as having access to good-quality information and making the quarantine voluntary has been shown to be associated with lower stress levels during the COVID-19 pandemic.^9,46^

### Physical and psychological QoL

Characteristics such as female gender, using medications daily, using controlled substances, having depressive symptoms, and negative forms of SRC were also associated with worse perceptions of their physical QoL in this study. These characteristics may somehow adversely affect physical QoL, since the latter depends on the ability to perform day-to-day activities, quality of sleep and ability to work.^46^ with regard to the psychological QoL domain, healthcare professionals and those indicating higher scores on PSRC reported significantly better scores, perhaps reflecting improved ability to cope more generally.

### Social relationships QoL

Not surprisingly, scores on the social relationships QoL domain were lower among participants who had a family member or friend with COVID-19 and among those who engaged in negative forms of SRC. The quarantine during the COVID-19 pandemic has limited personal contact with family and friends, adversely affected sexual activity, and has restricted other activities that are assessed in the social relationships QoL domain.^18^ In contrast, positive forms of SRC were associated with better scores on this domain, as reported in other studies.^23^ One explanation for the latter is that positive spiritual/religious behaviours involve maintaining a positive relationship with God as a way to cope and involves praying, meditating, and reflecting on God's power to help in stressful situations, including participation in the religious community resulting in increased social contacts (either in person or virtually).^40^

### Environmental QoL

Finally, healthcare professionals had better outcomes on environment QoL, whereas participants who had a friend or family member with COVID-19 had worse scores on this domain. Environment QoL involves financial resources, sense of freedom, a safe environment, acquisition of current information, and having an opportunity for recreation and leisure activities.^18^ Overall, healthcare professionals in Brazil are not subject to the quarantine because they must continue to care for people, and as noted earlier have higher incomes and better circumstances. Those who have had a friend or family member with COVID-19, however, may have been required to socially isolate themselves, which might compromise their environment QoL.

### Limitations

The present study has several limitations that may affect the generalizability and interpretation of results. First, is the cross-sectional nature of the study, which prevents causal inferences. Second, this was a convenience sample concentrated in the south-eastern part of Brazilian (78% of participants), making it difficult to generalise the findings across all of Brazil. Third, given the restrictions because of the pandemic, data collection was undertaken online, thereby limiting the sample to participants who had online access and were likely proficient with computers. Fourth, the use of ORs from logistic regression may have overestimated the associations, given the high prevalence of depression and anxiety, and we recommend that future studies of this type use methods that can estimate prevalence ratios, such as log-binomial modelling or Poisson regression. Fifth, we did not examine the living circumstances of participants to identify those who were living alone, which may have affected mental health and QoL. Finally, we cannot exclude residual confounding caused by unmeasured variables, such as social support, income or economic and political issues that were not assessed.

However, the study also has a number of strengths. The first is that our study, to the best of our knowledge, is one of the first to examine Brazilians’ mental health and QoL during the COVID-19 pandemic, and to use psychometrically valid measures to explore the relationships examined here. Furthermore, the data in this relatively large sample were examined using multivariate methods to identify independent risk factors for mental health problems during this critical time in Brazil.

### Implications

These findings may contribute to Brazilian healthcare policies during and after the COVID-19 pandemic. First, we have identified several factors associated with impaired mental health and poor QoL in Brazil during the first wave of the COVID-19 pandemic. These results suggest that Brazilians’ mental health and QoL may be seriously compromised during this pandemic. A high frequency of depressive and anxiety symptoms were identified, greatly surpassing figures obtained prior to the pandemic. Women, those with a friend or family member with COVID-19, those with significant depressive or anxiety symptoms, and individuals engaging in negative forms of SRC were more likely to have QoL impairments. In contrast, healthcare professionals, those who were more optimistic, and individuals engaged in positive forms of SRC reported better mental health and QoL. Future studies utilising a longitudinal design are needed to better understand the quarantine's impact on Brazilians’ mental health and QoL over time.

## Data Availability

The data are not publicly available because of privacy or ethical restrictions. However, the data that support the findings of this study are available from the corresponding author (L.M.V.), upon reasonable request.

## Supplementary material

For supplementary material accompanying this paper visit http://dx.doi.org/10.1192/bjo.2021.62. click here to view supplementary material
