# Supplementary material for: Factors associated with mental health and quality of life during the COVID-19 pandemic in Brazil
Source: BJPsych Open. 2021 May 14;7(3):e103. doi: 10.1192/bjo.2021.62 (PMC8129679; doi:10.1192/bjo.2021.62)
Supplement: Supplementary file 1 [file bjosup.zip › S2056472421000624sup001.docx]

**Supplementary Table 2. Effect of independent variables on quality of life domains (*N*=1156)**

| **Variables** | **WHOQOL-BREF** | **β (SE)** | **F** | **p-value** |
| --- | --- | --- | --- | --- |
|  | Physical health | -0.179 (0.131) | 1.880 | 0.171 |
| Sex | Psychological | -0.381 (0.128) | 8.886 | 0.003 |
|  | Social relationships | -0.266 (0.210) | 1.607 | 0.205 |
|  | Environment | -0.286 (0.038) | 3.823 | 0.051 |
|  |  |  |  |  |
|  | Physical health | -0.086 (0.096) | 0.810 | 0.368 |
| Marital status | Psychological | 0.078 (0.128) | 0.693 | 0.405 |
|  | Social relationships | 0.178 (0.154) | 1.341 | 0.247 |
|  | Environment | 0.018 (0.107) | 0.029 | 0.866 |
|  |  |  |  |  |
|  | Physical health | 0.0163 (0.127) | 1.659 | 0.198 |
| Healthcare professional | Psychological | 0.327 (0.124) | 6.993 | 0.008 |
|  | Social relationships | 0.053 (0.203 | 0.067 | 0.796 |
|  | Environment | 0.331 (141) | 5.458 | 0.020 |
|  |  |  |  |  |
|  | Physical health | -0.356 (0.156) | 5.208 | 0.023 |
| Daily Medication^a^ | Psychological | -0.174 (0.152) | 1.314 | 0.252 |
|  | Social relationships | -0.180 (0.250) | 0.518 | 0.472 |
|  | Environment | -0.141 (0.174) | 0.653 | 0.419 |
|  | Physical health | 0.362 (0.170) | 4.535 | 0.033 |
| Control Medication^a^ | Psychological | 0.038 (0.166) | 0.054 | 0.817 |
|  | Social relationships | -0.078 (0.273) | 0.081 | 0.776 |
|  | Environment | 0.213 (0.190) | 1.261 | 0.262 |
|  |  |  |  |  |
|  | Physical health | 0.271 (0.158) | 2.922 | 0.088 |
| Chronic illness | Psychological | 0.087 (0.155) | 0.316 | 0.574 |
|  | Social relationships | -0.162 (0.254) | 0.405 | 0.525 |
|  | Environment | -0.016 (0.177) | 0.008 | 0.928 |
|  |  |  |  |  |
|  | Physical health | -0.120 (0.133) | 0.812 | 0.368 |
| Friend/Family with COVID-19 | Psychological | -0.392 (0.185) | 3.796 | 0.010 |
|  | Social relationships | -0.358 (0.213) | 2.823 | 0.038 |
|  | Environment | -0.425 (0.148) | 8.195 | 0.004 |
|  |  |  |  |  |
|  | Physical health | -0.244 (0.015) | 267.068 | <0.001 |
| Depression | Psychological | -0.298 (0.016) | 312.906 | <0.001 |
|  | Social relationships | -0.189 (0.024) | 61.958 | <0.001 |
|  | Environment | -0.108 (0.017) | 41.881 | <0.001 |
|  |  |  |  |  |
|  | Physical health | -0.019 (0.019) | 1.438 | 0.231 |
| Anxiety | Psychological | -0.035 (0.015) | 5.204 | 0.023 |
|  | Social relationships | -0.001 (0.025) | 0.001 | 0.083 |
|  | Environment | -0.010 (0.018) | 0.294 | 0.588 |
|  |  |  |  |  |
|  | Physical health | 0.099 (0.052) | 3.527 | 0.061 |
| Positive SRC | Psychological | 0.360 (0.51) | 49.351 | <0.001 |
|  | Social relationships | 0.274 (0.084) | 10.585 | 0.001 |
|  | Environment | -0.042 (0.059) | 0.525 | 0.469 |
|  | Physical health | -0.532 (0.125) | 18.230 | <0.001 |
|  | Psychological | -0.776 (0.122) | 40.731 | <0.001 |
| Negative SRC | Social relationships | -0.651 (0.200) | 10.596 | 0.001 |
|  | Environment | -0.996 (0.139) | 51.245 | <0.001 |
|  |  |  |  |  |
|  | Physical health | 0.011 (0.016) | 0.203 | 0.652 |
| Optimism | Psychological | 0.023 (0.024) | 0.950 | 0.330 |
|  | Social relationships | 0.051 (0.039) | 1.741 | 0.187 |
|  | Environment | 0.028 (0.027) | 1.077 | 0.299 |
|  |  |  |  |  |
|  | Physical health | -0.010 (0.024) | 0.188 | 0.665 |
| Pessimism | Psychological | 0.017 (0.023) | 0.517 | 0.517 |
|  | Social relationships | -0.023 (0.038) | 0.348 | 0.348 |
|  | Environment | 0.017 (0.027) | 0.410 | 0.410 |
|  |  |  |  |  |
