# Supplementary material for: Factors associated with mental health and quality of life during the COVID-19 pandemic in Brazil
Source: BJPsych Open. 2021 May 14;7(3):e103. doi: 10.1192/bjo.2021.62 (PMC8129679; doi:10.1192/bjo.2021.62)
Supplement: Supplementary file 1 [file bjosup.zip › S2056472421000624sup002.docx]

| **Supplementary Table 1.** Univariate and multivariate analysis for crude and adjusted odds ratios for depressive symptoms and anxiety (n=1,156) | | | | |
| --- | --- | --- | --- | --- |
| **Variables** | **PHQ-9 (≥10)** | | **GAD-7 (≥10)** | |
|  | Unadjusted OR (95% CI), p | Adjusted OR (95% CI), p | Unadjusted OR (95% CI), p | Adjusted OR (95% CI), p |
| **Sex** |  |  |  |  |
| Male (reference category) |  |  |  |  |
| Female | - | - | 0.80 (0.61 - 1.05); 0.112 | 0.78 (0.59 - 1.04); 0.100 |
| **Age (mean)** | 0.99 (0.98 - 1.00); 0.043 | 0.99 (0.98 - 1.00); 0.146 | - | - |
| **Marital status** (single=reference) |  |  |  |  |
| Married / Living together | 0.80 (0.62 - 1.02); 0.083 | 0.97 (0.71 - 1.32); 0.856 | - | - |
| Divorced | 0.97 (0.64 - 1.48); 0.912 | 1.42 (0.85 - 2.35); 0.173 | - | - |
| **Health professionals** |  |  |  |  |
| No (reference) |  |  |  |  |
| Yes | 0.69 (0.54 - 0.89); 0.004 | 0.71 (0.55 - 0.93); 0.012 | - | - |
| **Chronic disease** |  |  |  |  |
| No (reference) |  |  |  |  |
| Yes | 1.22 (0.93 - 1.60); 0.150 | 1.23 (0.89 - 1.71); 0.195 | - | - |
| **Daily medication** |  |  |  |  |
| No (reference) |  |  |  |  |
| Yes | 1.17 (0.92 - 1.48); 0.182 | 0.95 (0.69 - 1.31); 0.791 | 1.23 (0.95 - 1.58); 0.108 | 1.18 (0.86 - 1.62); 0.295 |
| **Controlled medication** |  |  |  |  |
| No (reference) |  |  |  |  |
| Yes | 1.44 (1.09 - 1.90); 0.009 | 1.36 (0.96 - 1.92); 0.078 | 1.42 (1.06 - 1.91); 0.017 | 1.33 (0.92 - 1.93); 0.121 |
| **Physical activity^a,b^** |  |  |  |  |
| No (reference) |  |  |  |  |
| Yes | 0.79 (0.61 - 1.01); 0.068 | 0.81 (0.63 - 1.05); 0.124 | 0.80 (0.61 -1.05); 0.117 | 0.81 (0.61 - 1.08); 0.100 |
| **Admitted to health unit^a^** |  |  |  |  |
| No (reference) |  |  |  |  |
| Yes | - | 1.16 (0.79 - 1.70); 0.448 | - | - |
| **Clinical consultant^a^** |  |  |  |  |
| No (reference) |  |  |  |  |
| Yes | 1.26 (0.96 - 1.65); 0.087 | 1.26 (0.96 - 1.65); 0.087 | 1.20 (0.90 - 1.61); 0.201 | 1.12 (0.83 - 1.53); 0.437 |
|  |  |  |  |  |
| **Optimism (Mean)** | 0.92 (0.88 - 0.96); 0.001 | 0.94 (0.90 - 0.99); 0.018 | - | - |
|  |  |  |  |  |
| **Positive SRC (Mean)** | - | - | 1.11 (0.99 - 1.24); 0.057 | 1.00 (0.89 - 1.13); 0.912 |
|  |  |  |  |  |
| **Negative SRC (Mean)** | 2.13 (1.63 - 2.80); <0.001 | 2.15 (1.65 - 2.80);<0.001 | 2.46 (1.90 - 3.18); <0.001 | 2.53 (1.93-3.31); <0.001 |
|  |  |  |  |  |
| **Social isolation in days (Mean)** | - | - | 0.99 (0.98 - 0.99); 0.004 | 0.99 (0.98 - 0.99); 0.004 |
|  |  |  |  |  |

OR: odds ratio; p: p-value
